# Efficacy of Vitamin D Supplements in Treatment of Acute Respiratory Infection: A Meta-analysis for Randomized Controlled Trials

**DOI:** 10.3390/nu14061144

**Published:** 2022-03-08

**Authors:** Herim Cho, Seung-Kwon Myung, Hae-Eun Cho

**Affiliations:** 1 Department of Medicine, College of Medicine, Ewha Womans University, Seoul 07804, Korea; herimcho0725@gmail.com (H.C.); puerile97@naver.com (H.-E.C.); 2 Department of Cancer Biomedical Science, National Cancer Center Graduate School of Cancer Science and Policy, Goyang 10408, Korea; 3 Cancer Epidemiology Branch, Division of Cancer Data Science, National Cancer Center Research Institute, Goyang 10408, Korea; 4 Department of Family Medicine and Center for Cancer Prevention and Detection, National Cancer Center Hospital, Goyang 10408, Korea

**Keywords:** vitamin D supplements, acute respiratory infections, randomized-controlled trial, meta-analysis

## Abstract

Background: Recent randomized controlled trials (RCTs) have reported inconsistent findings regarding the efficacy of vitamin D supplementation in the treatment of acute respiratory infections (ARIs). This study aimed to investigate the efficacy of vitamin D supplementation in the treatment of ARIs using a meta-analysis of RCTs. Methods: PubMed, EMBASE, and the Cochrane Library were searched for relevant articles in June 2021. Two of the authors independently assessed the eligibility of the trials. Results: Out of 390 articles retrieved from the databases, we included 18 RCTs, which involved 3648 participants, with 1838 in an intervention group and 1810 in a control group in the final analysis. In the meta-analysis of all the trials, vitamin D supplements had a beneficial effect in the treatment of ARIs (relative risk (RR) = 1.07; 95% confidence interval (CI), 1.01–1.13; I^2^ = 66.9%). Publication bias was observed in the funnel plot. In the subgroup meta-analysis of high-quality RCTs, no significant efficacy of vitamin D supplements was found (RR = 1.02; 95% CI, 0.98–1.06; I^2^ = 24.0%). Although statistically significant changes of 7% in the treatment effects were observed, they are not considered as clinically substantial ones. Conclusions: The current meta-analysis suggests that vitamin D supplements are not clinically effective in the treatment of ARIs.

## 1. Introduction

Acute respiratory infections (ARIs) can be classified into upper respiratory tract infections (URIs) and lower respiratory tract infections (LRIs) [1,2]. URIs include common cold, sinusitis, pharyngitis, epiglottis, and laryngotracheitis, and LRIs include pneumonia, bronchitis, and bronchiolitis [3]. URIs in particular have continued to be a significant economic and social burden in the community, with young children suffering, on average, 6–8 and adults 2–4 URIs per year [4,5]. Because most URIs are self-limiting, the treatments focus primarily on symptom relief [2,4,5]. The treatment modalities for URIs include symptomatic therapy, non-steroidal anti-inflammatory drugs, and first-generation antihistamines, while the efficacy of zinc, vitamin C, or echinacea extracts remains inconclusive [5]. In 2019, LRIs were ranked second among the top 10 causes of disability-adjusted life-years (DALYs) in children under the age of 10 and ranked within the top 10 in people aged 75 and older [6]. The evidence-based recommendations for the treatment of LRIs involve antibiotics, intravenous fluids, ventilation support, and others [7].

A hormonally active form of vitamin D (a.k.a., calcitriol, 1,25(OH)2D) is essential for bone and mineral homeostasis [8]. In addition, vitamin D plays an essential role in immune function by increasing anti-viral defenses [9,10]. Vitamin D also increases antiviral defenses via cathelicidin (in the form of 37 amino acid cationic peptide (LL-37)) and innate interferon pathways and suppresses the receptors that induce inflammation [11,12]. In an animal study, after viral infection in 25(OH)D3-fed mice, the proinflammatory cytokines interleukin-5 (IL-5) and Interferon gamma (IFN-γ) were significantly downregulated, indicating that 25(OH)D3 decreases the production of inflammatory cytokines and viral replication [13].

Previous randomized controlled trials (RCTs) have shown inconsistent findings regarding the efficacy of vitamin D supplements in the treatment of ARIs [14,15,16,17,18,19,20,21,22,23,24,25,26,27,28,29,30,31]. Several RCTs reported that vitamin D supplements had a beneficial effect [14,19,21,28,29,30], while others reported no effect [15,16,17,18,20,22,23,24,25,26,27,31]. Several meta-analyses have been published on the efficacy of vitamin D supplements in the treatment of pulmonary tuberculosis (TB), pneumonia, and COVID-19 [32,33,34,35,36,37,38,39,40,41,42]. However, no meta-analysis has been published for the efficacy of vitamin D supplements in the treatment of comprehensive ARIs.

In the current study, we investigated whether vitamin D supplementation is efficacious in the treatment of ARIs by using a meta-analysis of RCTs based on various factors affecting the outcomes.

## 2. Methods and Materials

### 2.1. Literature Search Strategy

Eligible studies were identified by searching PubMed, EMBASE, and the Cochrane Library in June 2021. We combined the National Library of Medicine (NLM) Medical Subject Heading (MeSH) terms and a wide range of free-text terms as search terms in order to locate as many relevant articles as possible. We used a PICO framework to determine search terms related to the topic of this study as follows: P for population is ‘patients with ARIs’; I for intervention is ‘vitamin D supplements’; C for comparison is ‘placebos’; and O for outcome is ‘treatment effects or efficacy’. We restricted the study design to an RCT for the current study. Thus, by using Boolean operators for all the determined MeSH and free-text terms, we created a combination of search terms as follows: ‘vitamin D and respiratory tract infections and randomized controlled trial’. We further reviewed the reference lists from the identified articles to find relevant studies not identified through this search strategy. We limited the search to the articles that were written in English.

### 2.2. Selection Criteria

We included RCTs that reported the efficacy of vitamin D supplements in the treatment of ARIs using outcome measures with dichotomous variables. Vitamin D supplementation included both oral or parenteral administration. If studies with the same data were published in more than one article, we included a more comprehensive article in the current study.

### 2.3. Selection of Relevant Studies

Based on the above described selection criteria, two authors (H. Cho and H.-E Cho) independently selected appropriate studies retrieved from the databases and bibliographies. Disagreements on the selection among the authors were resolved by consultation of the third author.

### 2.4. Assessment of Methodological Quality

We assessed the risk of bias using the Cochrane Risk of Bias Tool [43] and the Jadad scale [44]. In the Cochrane Risk of Bias Tool, trials with a low risk of bias in more than the average number of items across all the trials were considered as having overall low risk of bias in the current study. Because the mean score of the 18 trials included in this study was 5.5, those with a score of 5 or lower were considered as having low quality, and the remaining trials with 6 or higher were considered as having high quality. In addition, because the mean score of the Jadad scale for the 18 trials was 4.4, trials with a score of 4 or lower were considered as having low quality, and the remaining trials with a score of 5 were considered as having high quality. Two authors (H. Cho and H.-E. Cho) assessed the methodological quality. In addition, we assessed overall certainty of evidence by using the GRADE methodology (GRADEpro, GDT: GRADEpro Guideline Development Tool (www.gradepro.org, accessed on 28 February 2022, McMaster University and Evidence Prime, Hamilton, ON, Canada).

### 2.5. Main and Subgroup Analyses

We investigated the efficacy of vitamin D supplements in the treatment of ARIs in the main analysis. The main outcomes included sputum conversion, survival rate, therapeutic success, and no need for intensive care unit (ICU) admission. Therapeutic success included hospital discharge, resolution of chest radiograph infiltrate, and no lower chest retraction.

Subgroup analyses were performed according to the following factors: type of outcome (sputum conversion, survival rate, therapeutic success, or no need for ICU admission), type of disease (pulmonary TB, pneumonia, or COVID-19), total dosage of vitamin D supplementation (less than 350,000 IU vs. more than 350,000 IU), type of study design (randomized, double-blind, placebo-controlled type (RDBPCT) vs. open-label, randomized controlled trial (OLRCT)), methodological quality (high vs. low), duration of treatment (less than 12 weeks vs. 12 weeks or longer), supply source for supplements (pharmaceutical industry vs. no pharmaceutical industry), age (15 years or older vs. younger than 5 years), route of administration (oral vs. injection), type of continent (Asia, Europe, Africa, Oceania, or South America), and number of participants (less than 200 vs. 200 or more).

### 2.6. Statistical Analysis

We calculated a pooled relative risk (RR) with its 95% confidence interval (CI) using values in cells of a 2 × 2 table. I^2^ was used in order to test heterogeneity across the studies, which is the percentage of total variation across study results [45].

I^2^ was calculated as follows:

I^2^ = 100% × (Q − df)/Q,

where Q denotes Cochran’s heterogeneity statistic, and df does degrees of freedom. I^2^ ranges from 0% (no heterogeneity) to 100% (maximal heterogeneity). An I^2^ > 50% was considered as having substantial heterogeneity [45]. We used a random-effects model meta-analysis on the basis of the DerSimonian and Laird method because individual studies were performed in different populations.

In order to estimate the publication bias, the Begg’s funnel plot and Egger’s test were used in the current study. If there is publication bias, the Begg’s funnel plot is asymmetrical, or the Egger’s test shows a p-value less than 0.05. For the statistical analysis, we used the Stata SE version 17.0 software package (StataCorp, College Station, TX, USA).

## 3. Results

### 3.1. Identification of Relevant Studies

**Figure 1:**
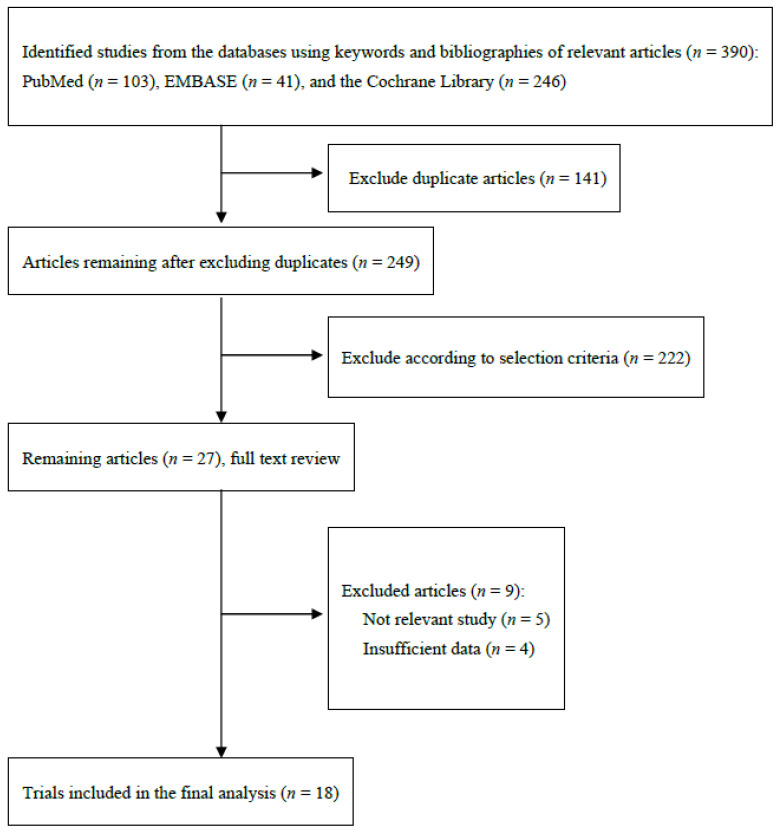
Flow diagram for identification of relevant clinical trials.

A flow diagram in Figure 1 shows how we identified relevant RCTs. A total of 390 articles were retrieved from the core databases: PubMed, EMBASE, and the Cochrane Library. After excluding 141 duplicated articles, two authors independently reviewed the title and abstract of each article and excluded 222 articles that did not meet the selection criteria. After reviewing the full texts of the 27 articles, we additionally excluded 9 articles because of the following reasons: not relevant topic (*n* = 5) or insufficient data (*n* = 4).

### 3.2. General Characteristics of Trials

Table 1 shows the general characteristics of all the RCTs included in the final analysis. The 18 trials included a total of 3648 participants, with 1838 participants in the intervention group and 1810 participants in the control group. The number of participants ranged between 46 and 496. The mean age of the participants ranged between 12 months and 62 years. The countries where the studies were conducted are as follows: India (*n* = 4), Indonesia (*n* = 2), Egypt (*n* = 2), UK (*n* = 1), New Zealand (*n* = 1), Brazil (*n* = 1), Spain (*n* = 1), Pakistan (*n* = 1), Mongolia (*n* = 1), Iran (*n* = 1), Bangladesh (*n* = 1), Georgia (*n* = 1), and Guinea-Bissau (*n* = 1). The supplementation and follow-up periods ranged between 5 days and 12 months. The year of publication of the studies ranged from 2006 to 2021.

Fifteen studies were RDBPCTs, and three were OLRCTs. Sixteen RCTs used oral administration for vitamin D supplements, and two used intramuscular injection.

The dosage of vitamin D used in each trial was as follows: 100,000, 200,000, or 300,000 IU once; 600, 1000, 5000, or 10,000 IU daily; 100,000 or 140,000 IU every 2 weeks; 50,000 or 600,000 IU two times over a month; 100,000 IU three times over 8 months; 50,000 IU seven times over 16 weeks; or 32,000 IU once plus 16,000 IU twice a week plus 16,000 weekly.

Out of 18 trials, 16 trials were funded by public/governmental organizations or independent scientific foundations, while the remaining ones did not report their funding source. Regarding the source of supplements, six trials were provided by a pharmaceutical company, two paid for them, and 10 did not mention their funding source.

### 3.3. Main Findings

**Figure 2:**
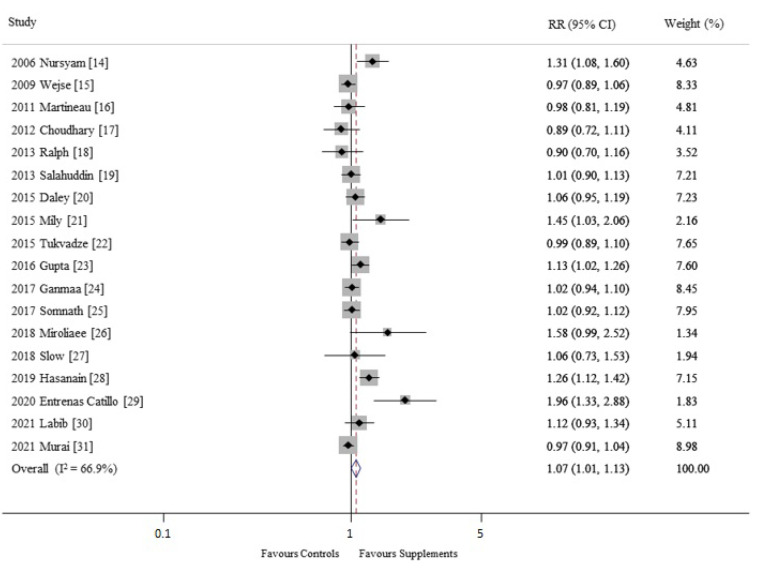
Efficacy of vitamin D supplements in treatment of acute respiratory infections in a meta-analysis of randomized controlled trials (*n* = 18) [14,15,16,17,18,19,20,21,22,23,24,25,26,27,28,29,30,31]. RR, relative risk; CI, confidence interval.

**Figure 3:**
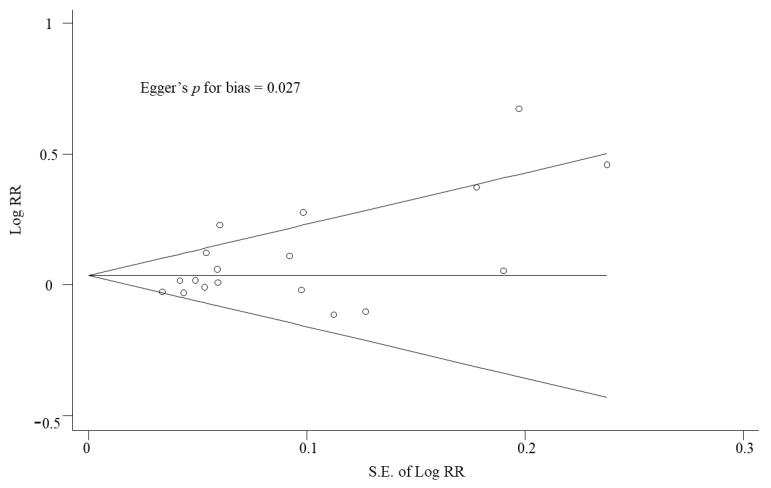
Begg’s funnel plots and Egger’s test for identifying publication bias. RR, relative risk; S.E., standard error.

In the random-effects meta-analysis of all 18 trials, vitamin D supplementation had a beneficial effect in the treatment of ARIs (RR, 1.07; 95% CI, 1.01–1.13, I^2^ = 66.9%) (Figure 2). The Begg’s funnel plot showed asymmetry in all the 18 trials, and the Egger’s test showed that P for bias was 0.027 (Figure 3).

### 3.4. Assessment of Methodological Quality of Studies

There were 12 high-quality trials and 6 low-quality trials according to the Cochrane Risk of Bias Tool (Table 2), and 11 high-quality trials and 7 low-quality trials according to the Jadad scale (Table 3). Overall, very low quality of evidence shows that vitamin D supplements have a beneficial efficacy in the treatment of ARIs, because of very serious risk of bias, serious inconsistency and indirectness, and strongly suspected publication bias (Table 4).

### 3.5. Subgroup Meta-Analysis by Various Factors

Table 5 shows the efficacy of vitamin D supplementation in the treatment of the ARIs in subgroup meta-analysis according to various factors. Overall, no significant efficacy was found in the subgroup meta-analysis by type of vitamin D dosage (total dose < 350,000 IU vs. total dose > 350,000 IU), assessment of treatment efficacy (sputum conversion, survival rate, therapeutic success, or no need for ICU admission), type of disease (pulmonary TB, pneumonia, or COVID-19), type of continent (Asia, Europe, Africa, Oceania, or South America), type of study design (RDBPCT vs. OLRCT), methodological quality (high vs. low), duration of treatment (<12 weeks vs. ≥12 weeks), supply source for supplements (pharmaceutical industry vs. no pharmaceutical industry), and number of participants in each trial (less than 200 vs. 200 or more).

## 4. Discussion

The current meta-analysis of 18 RCTs showed that the use of vitamin D supplements showed a statistically significant efficacy in the treatment of ARIs (RR, 1.07; 95% CI, 1.01–1.13, I^2^ = 66.9%). However, in general, because clinically significant efficacy requires a substantial difference in the outcomes between the intervention and control groups, its efficacy of 7% in the treatment of ARIs is considered trivial.

Several biological mechanisms are possible for the beneficial effects of vitamin D in the treatment of ARIs. First, vitamin D could be a direct regulator of antimicrobial innate immune responses [46,47]. The innate response can be triggered by the activation of toll-like receptors (TLRs) of polynuclear cells, macrophages, monocytes, and epithelial cells [47]. Because multiple TLRs both affect and are affected by vitamin D receptor (VDR) stimulation, vitamin D3 (1,25(OH)2D3) could inhibit TLR2 and TLR4 protein expression [9,12]. Despite the fact that TLRs play an important role in the activation of protective immune responses, research suggests that the extensive release of TLR-triggered pro-inflammatory mediators might be harmful to the host organism [12]. When TLRs are activated, vitamin D3 interferes with this process and also promotes the production of cathelicidin, an antibacterial peptide [46,47]. Second, vitamin D has inhibitory effects on the adaptive immune system [46,48]. During the adaptive immune response, to specifically combat the source of the antigen presented to cells such as dendritic cells and macrophages, T and B lymphocytes produce cytokines and immunoglobulins, respectively [48]. Vitamin D suppresses T helper type 1 (Th-1) cell proliferation, resulting in lower production of interferon gamma (INF-a) and interleukin-2 (IL-2) [9,49,50,51]. Lower levels of circulating cytokines result in less antigens presentation by dendritic cells, as well as less T lymphocyte recruitment and proliferation [9,51]. In addition, vitamin D inhibits the differentiation of B cell precursors into plasma cells, as well as the production of immunoglobulin [48,49]. Lastly, vitamin D protects the lung by increasing alveolar type II (ATII) cell proliferation, decreasing epithelial cell apoptosis, and inhibiting transforming growth factor-β (TGF-β) [52]. Vitamin D stimulates primary human ATII cell proliferation via the phophatidylinositol-3-kinase and protein kinase B (PI3K/AKT) signaling pathway and activation of VDR [52].

However, findings from previous RCTs are inconsistent on the effects of vitamin D supplements in the treatment of ARIs. Since 2018, several meta-analyses on this topic have been published: six meta-analyses on the efficacy of vitamin D supplements in the treatment of pulmonary TB, three on the treatment of pneumonia, and two on the treatment of COVID-19. Regarding pulmonary TB, two meta-analyses [39,40] reported that a significant accelerated sputum conversion was observed, three [33,34,35] reported that there was no significant benefit because the time to sputum conversion was not shortened or the proportion of sputum smear and culture conversion was not increased, and one [32] reported that the time to sputum smear and culture conversion did not improve, whereas the proportion of sputum smear and culture conversion increased. Among the three meta-analyses [36,37,38] about the treatment of pneumonia, two of them [36,38] reported that vitamin D supplementation had no marked efficacy in the treatment of pneumonia, and the remaining one [37] reported that it was uncertain whether vitamin D supplementation was effective because the results were imprecise. Among the two meta-analyses [41,42] about the treatment of COVID-19, one [41] reported a statistically lower ICU requirement in patients with vitamin D supplementation, while the other one [42] reported no significant efficacy on major health-related outcomes in patients with COVID-19.

Unlike the previous meta-analyses, we investigated all types of ARIs and performed comprehensive subgroup meta-analyses according to various factors such as type of study quality, disease, and vitamin D dosage. The subgroup meta-analyses by study quality in our study showed interesting findings. High-quality studies showed no significant efficacy of vitamin D in the treatment of ARIs, while low-quality studies showed significant beneficial efficacy. Cochrane reviews discourage the use of scales or scores for assessing quality or risk of bias due to difficulties in justifying assigning weights to different items in the scale and unreliable assessments of validity by the scales [43]. Despite these limitations, we believe that it would be helpful to investigate whether there is any discrepancy in the results according to study quality. In addition, while heterogeneity was 24.0% across the trials with a low risk of bias, it was 81.1% across the trials with a high risk of bias. We think that trials with a low risk of bias in six or more items are more likely to show the results closer to the truth.

The current meta-analysis has several strengths. First, this study is considered as the first meta-analysis that investigated the efficacy of vitamin D supplements in the treatment of ARIs. Second, we only included RCTs, which gives us a higher level of evidence than observational studies in general. Third, we conducted subgroup meta-analyses for important factors that could affect individual results, such as the methodological quality, type of disease, and dosage of vitamin D supplements.

There are several limitations in the current study. First, even though we included 18 studies with 3648 study participants, most of these had a smaller sample size, of less than 300 participants [1,3,4,5,6,7,8,9,12,13,14,16,17]. Thus, further larger trials are warranted to confirm our findings on the efficacy of vitamin D for ARIs. Second, five [15,19,25,26,30] out of 18 RCTs were not designed specifically to investigate the efficacy in the treatment of ARIs as primary outcomes. For example, as a primary outcome, Wejse et al. [15] used reduction in a clinical severity score, and Miroliaee et al. [26] used the effect of vitamin D administration on the selected markers (IL-6, C-reactive protein, and plasma level of vitamin D). In general, findings in the secondary endpoint might be due to chance, because the trial was not designed specifically to assess this. Lastly, publication bias was observed in the Begg’s funnel plots and the Egger’s test.

## 5. Conclusions

In conclusion, regarding the efficacy of vitamin D supplements in the treatment of ARIs, even though a statistical significance was observed in the main meta-analysis, there was no sufficient clinical significance, with only 7% of improvement in efficacy. Additionally, no significant efficacy in the subgroup meta-analysis of high-quality studies and the existence of publication bias in the main analysis support our conclusion. However, further large RCTs are warranted to confirm our findings on the efficacy of vitamin D supplements in the treatment of ARIs.

## Figures and Tables

**Table 1:** Characteristics of studies included in the final analysis (*n* = 18).

	Study	Country	Study Design	Participants (Average Age, y; Women, %)	Supplementation Period (Follow-Up Period)	Intervention vs. Control	Main Outcome Measures	No. of Participants in Remission/No. of Participants	
								Supplement Group	Placebo Group
1	2006, Nursyam et al. [14]	Indonesia	RDBPCT	67 patients with moderately advanced pulmonary TB (31; 41)	6 w (6 w)	Vitamin D (0.25 mg/d) vs. placebo (PO)	Sputum conversion	34/34	25/33
2	2009, Wejse et al. [15]	Guinea-Bissau	RDBPCT	365 patients with TB (37; 40)	8 m (12 m)	Vitamin D (100,000 IU 3 times) vs. placebo (PO)	Survival rate	157/187	154/178
3	2011, Martineau et al. [16]	UK	RDBPCT	108 patients with pulmonary TB (30; 22)	56 d (56 d)	Vitamin D (100,000 IU/2 w 4 times) vs. placebo (PO)	Sputum conversion	41/52	45/56
4	2012, Choudhary et al. [17]	India	RDBPCT	200 patients with severe pneumonia (14 months; 40)	5 d (5 d)	Vitamin D (1000 IU or 2000 IU/d) vs. placebo (PO)	Discharged within 120 h	58/100	65/100
5	2013, Ralph et al. [18]	Indonesia	RDBPCT	155 patients with pulmonary TB (28; 35)	2 m (1 m)	Vitamin D (50,000 IU/m) vs. placebo (PO)	Sputum conversion	44/75	52/80
6	2013, Salahuddin et al. [19]	Pakistan	RDBPCT	259 patients with pulmonary TB (28; 46)	2 m (3 m)	Vitamin D (600,000 IU/m) vs. placebo (IM)	Sputum conversion	108/132	103/127
7	2015, Daley et al. [20]	India	RDBPCT	198 patients with pulmonary TB (42; 23)	6 w (6 m)	Vitamin D (2.5 mg /2 w) vs. placebo (PO)	Sputum conversion	87/99	82/99
8	2015, Mily et al. [21]	Bangladesh	RDBPCT	126 patients with pulmonary TB (27; 38)	1 m (1 m)	Vitamin D (5000 IU/d) vs. placebo (PO)	Sputum conversion	38/62	27/64
9	2015, Tukvadze et al. [22]	Georgia	RDBPCT	192 patients with pulmonary TB (33; 36)	4 m (4 m)	Vitamin D (50,000 IU 3 times/w for 8 w, 50,000 IU/2 w for additional 8 w) vs. placebo (PO)	Sputum conversion	85/97	84/95
10	2016, Gupta et al. [23]	India	RDBPCT	309 patients with pneumonia (12 months; 30)	once (about 30 h)	Vitamin D (100,000 IU once at enrollment) vs. placebo (PO)	Time to resolution of severe pneumonia	133/153	120/156
11	2017, Ganmaa et al. [24]	Mongolia	RDBPCT	352 patients with pulmonary TB (33; 67)	2 m (2 m)	Vitamin D (140,000 IU/2 w) vs. placebo (PO)	Sputum conversion	152/174	153/178
12	2017, Somnath et al. [25]	India	OLRCT	154 patients with acute lower respiratory infection (13 months; 32)	once (4.5–9 d)	Vitamin D (100,000 IU once) vs. placebo (PO)	No need for PICU transfer	72/78	69/76
13	2018, Miroliaee et al. [26]	Iran	RDBPCT	46 patients with ventilator-associated pneumonia (57; 43)	once (1 m)	Vitamin D (300,000 IU once) vs. placebo (IM)	Survival rate	19/24	11/22
14	2018, Slow et al. [27]	New Zealand	RDBPCT	117 patients with community-acquired pneumonia (62; 37)	once (6 w)	Vitamin D (200,000 IU once) vs. placebo (PO)	Complete resolution of chest radiograph infiltrate	30/60	27/57
15	2019, Hasanain et al. [28]	Egypt	OLRCT	496 patients with TB (32; 56)	4 m (4 m)	Vitamin D (600 IU/d) vs. placebo (PO)	Negative sputum culture	194/249	153/247
16	2020, Entrenas Castillo et al. [29]	Spain	OLRCT	76 patients with COVID-19 (53; 41)	n.a. (n.a.)	Vitamin D (32,000 IU at admission, 16,000 IU on day 3 and day 7, and 16,000 IU/w) vs. placebo (PO)	Not requiring ICU admission	49/50	13/26
17	2021, Labib et al. [30]	Egypt	RDBPCT	191 patients with pneumonia (2; 29)	once (n.a.)	Vitamin D (100,000 IU once) vs. placebo (PO)	Survival rate	70/93	66/98
18	2021, Murai et al. [31]	Brazil	RDBPCT	337 patients with COVID-19 (56; 43)	once (about 7 d)	Vitamin D (200,000 IU once) vs. placebo (PO)	Survival rate	110/119	112/118

n.a., not available; RDBPCT, randomized, double-blind, placebo-controlled trial; OLRCT, open-label, randomized, controlled trial; y, years; w, weeks; m, months; d, days; h, hours; TB, tuberculosis; COVID-19, coronavirus disease-19; IU, international unit; PO, per os administration; IM, intramuscular injection; PICU, pediatric intensive care unit; ICU, intensive care unit.

**Table 2:** Summary of risk of bias assessment for randomized, double-blind, placebo-controlled trials based on the Cochrane Risk of Bias Tool (mean score = 5.5).

Study	Random Sequence Generation	Allocation Concealment	Blinding of Participants and Personnel	Blinding of Outcome Assessment	Incomplete Outcome Data	Selective Reporting	Other Bias	No. of Low Risk of Bias
2006, Nursyam et al. [14]	Unclear	Unclear	Low	Unclear	Unclear	Low	Low	3
2009, Wejse et al. [15]	Low	Low	Low	Low	High	Low	Low	6
2011, Martineau et al. [16]	Low	Low	Low	Low	High	Low	Low	6
2012, Choudhary et al. [17]	Low	Low	Low	Unclear	Unclear	Low	Low	5
2013, Ralph et al. [18]	Low	Low	Low	Low	High	Low	Low	6
2013, Salahuddin et al. [19]	Low	Unclear	Low	Low	Low	Low	Low	6
2015, Daley et al. [20]	Low	Low	Low	Low	High	Low	Low	6
2015, Mily et al. [21]	Low	Low	Low	Low	High	Low	Low	6
2015, Tukvadze et al. [22]	Low	Low	Low	Low	Low	Low	Low	7
2016, Gupta et al. [23]	Low	Low	Low	Low	Low	Low	Low	7
2017, Ganmaa et al. [24]	Low	Low	Low	Low	Low	Low	Low	7
2017, Somnath et al. [25]	Unclear	High	High	High	Low	Low	Low	3
2018, Miroliaee et al. [26]	Low	Unclear	Unclear	Low	Low	Low	Low	5
2018, Slow et al. [27]	Low	Low	Low	Low	High	Low	Low	6
2019, Hasanain et al. [28]	Low	High	High	High	Low	Low	Low	4
2020, Entrenas Castillo et al. [29]	Low	High	High	High	High	Low	Low	3
2021, Labib et al. [30]	Low	Low	Low	Low	Low	High	Low	6
2021, Murai et al. [31]	Low	Low	Low	Low	Low	Low	Low	7

**Table 3:** Methodological quality of trials based on the Jadad Scale (*n* = 18).

	Source	Randomization	Description of Randomization Methods	Double-Blind	Using Identical Placebo	Follow-Up Reporting	Total Score
1	2006, Nursyam et al. [14]	1	0	1	1	0	3
2	2009, Wejse et al. [15]	1	1	1	1	1	5
3	2011, Martineau et al. [16]	1	1	1	1	1	5
4	2012, Choudhary et al. [17]	1	1	1	1	0	4
5	2013, Ralph et al. [18]	1	1	1	1	1	5
6	2013, Salahuddin et al. [19]	1	1	1	0	1	4
7	2015, Daley et al. [20]	1	1	1	1	1	5
8	2015, Mily et al. [21]	1	1	1	1	1	5
9	2015, Tukvadze et al. [22]	1	1	1	1	1	5
10	2016, Gupta et al. [23]	1	1	1	1	1	5
11	2017, Ganmaa et al. [24]	1	1	1	1	1	5
12	2017, Somnath et al. [25]	1	1	0	0	1	3
13	2018, Miroliaee et al. [26]	1	1	1	1	1	5
14	2018, Slow et al. [27]	1	1	1	1	1	5
15	2019, Hasanain et al. [28]	1	1	0	0	1	3
16	2020, Entrenas Castillo et al. [29]	1	1	0	0	1	3
17	2021, Labib et al. [30]	1	1	1	1	1	5
18	2021, Murai et al. [31]	1	1	1	1	1	5

**Table 4:** Certainty of evidence and summary of findings based on the GRADE methodology.

Certainty Assessment							No. of Patients		Effect		Certainty
No. of Studies	Study Design	Risk of Bias	Inconsistency	Indirectness	Imprecision	Other Considerations	Vitamin D	Placebo	RR (95% CI)	RD (95% CI)	
Outcome: Treatment efficacy (sputum conversion, survival rate, therapeutic success, resolution of chest radiograph infiltrate, and hospital discharge)											
18	Randomized controlled trials	Serious ^a^	Serious ^b^	Serious ^c^	Not serious ^d^	Publication bias strongly suspected ^e^	1481/1838 (80.6%)	1361/1810 (75.2%)	RR 1.07 (1.01 to 1.13)	53 more per 1000 (from 8 more to 98 more)	⨁◯◯◯ Very low

RR, relative risk; CI, confidence interval; RD, risk difference; CI, confidence interval; ⨁◯◯◯, 1 positive out of 4 positive circles. ^a^ Most information is from trials at low or unclear risk of bias; ^b^ I^2^ for heterogeneity is 66.9%; ^c^ outcomes are diverse; ^d^ the sample size is 3648, and 95% CI excludes 1; ^e^ *p*-value for publication bias by Egger’s test is 0.027.

**Table 5:** Efficacy of vitamin D in the treatment of the upper respiratory infections in random-effect meta-analyses by various factors.

Factor	No. of Trials	Summary RR (95% CI)	Heterogeneity, I^2^
All *	18 [14,15,16,17,18,19,20,21,22,23,24,25,26,27,28,29,30,31]	1.07 (1.01–1.13)	66.9%
Dosage			
Low dose (total dose < 350,000 IU)	12 [15,17,18,21,23,25,26,27,28,29,30,31]	1.09 (1.00–1.20)	76.3%
High dose (total dose ≥ 350,000 IU)	6 [14,16,19,20,22,24]	1.03 (0.98–1.10)	29.2%
Assessment of treatment efficacy			
Sputum conversion	8 [14,16,18,19,20,21,22,24]	1.04 (0.97–1.11)	41.5%
Increased survival rate	4 [15,26,30,31]	1.02 (0.92–1.13)	60.1%
Therapeutic success	4 [17,23,27,28]	1.11 (0.97–1.27)	60.7%
No need for ICU	2 [25,29]	1.39 (0.60–3.22)	94.4%
Type of disease			
Pulmonary TB	10 [14,15,16,18,19,20,21,22,24,28]	1.06 (0.99–1.14)	65.2%
Pneumonia	6 [17,23,25,26,27,30]	1.07 (0.98–1.17)	35.0%
COVID-19	2 [29,31]	1.36 (0.54–3.43)	95.5%
Age			
≥15 years **	14 [14,15,16,18,19,20,21,22,24,26,27,28,29,31]	1.08 (1.00–1.16)	72.8%
<5 years	4 [17,23,25,30]	1.05 (0.97–1.15)	37.7%
Route of administration			
Oral **	16 [14,15,16,17,18,20,21,22,23,24,25,27,28,29,30,31]	1.07 (1.00–1.13)	68.6%
Injection	2 [19,26]	1.20 (0.77–1.86)	72.4%
Type of continent			
Asia	10 [14,17,18,19,20,21,23,24,25,26]	1.06 (0.99–1.14)	50.6%
Europe	3 [16,22,29]	1.17 (0.86–1.59)	85.6%
Africa	3 [15,28,30]	1.11 (0.92–1.33)	85.8%
Oceania	1 [27]	1.06 (0.73–1.53)	n.a.
South America	1 [31]	0.97 (0.91–1.04)	n.a.
Study design			
RDBPCT	15 [14,15,16,17,18,19,20,21,22,23,24,26,27,30,31]	1.03 (0.99–1.09)	45.3%
OLRCT	3 [25,28,29]	1.28 (0.96–1.71)	90.7%
Methodological quality			
High-quality (Risk of Bias ≥ 6)	12 [15,16,18,19,20,21,22,23,24,27,30,31]	1.02 (0.98–1.06)	24.0%
Low-quality (Risk of Bias < 6) *	6 [14,17,25,26,28,29]	1.22 (1.02–1.42)	81.1%
High-quality (Jadad score = 5)	11 [15,16,18,20,21,23,24,26,27,30,31]	1.04 (0.98–1.10)	46.0%
Low-quality (Jadad score ≤ 4)	7 [14,17,19,22,25,28,29]	1.11 (0.99–1.26)	79.8%
Duration of treatment			
<12 weeks	11 [14,16,17,18,21,23,24,26,27,30,31]	1.06 (0.98–1.15)	57.6%
≥12 weeks	5 [15,19,20,22,28]	1.05 (0.96–1.15)	73.7%
Not mentioned	2 [25,29]	1.39 (0.60–3.22)	94.4%
Supply source for supplements			
Pharmaceutical industry	6 [15,16,20,23,25,31]	1.02 (0.97–1.07)	33.8%
No pharmaceutical industry	2 [18,21]	1.13 (0.70–1.80)	79.3%
Not mentioned *	10 [14,17,19,22,24,26,27,28,29,30]	1.12 (1.01–1.24)	72.5%
No. of participants in each trial			
<200	11 [14,16,18,21,22,25,26,27,29,30,31]	1.10 (1.00–1.22)	71.8%
≥200	7 [15,17,19,20,23,24,28]	1.05 (0.98–1.13)	66.2%

n.a., not available; * Statistically significant; ** Marginally significant. RDBPCT, randomized, double-blind, placebo-controlled trial; OLRCT, open-label, randomized controlled trial.

## Data Availability

Not applicable.
